# Zinc Cream and Reliability of Tuberculosis Skin Testing

**DOI:** 10.3201/eid1307.070227

**Published:** 2007-07

**Authors:** V. Bhargavi Rao, Tom F. Pelly, Robert H. Gilman, Lilia Cabrera, Jose Delgado, Giselle Soto, Jon S. Friedland, A. Roderick Escombe, Robert E. Black, Carlton A. Evans

**Affiliations:** * Imperial College London, London, UK; † London School of Hygiene and Tropical Medicine, London, UK; ‡ Asociación Benéfica Prisma, Lima, Peru; § Universidad Peruana Cayetano Heredia, Lima, Peru; ¶ Johns Hopkins Bloomberg School of Public Health, Baltimore, Maryland, USA

**Keywords:** tuberculosis, zinc, diagnosis, tuberculin, purified protein derivative, nutrition, dispatch

## Abstract

In 50 healthy Peruvian shantytown residents, zinc cream applied to tuberculosis skin-test sitescaused a 32% increase in induration compared with placebo cream. Persons with lower plasma zinc had smaller skin-test reactions and greater augmentation with zinc cream. Zinc deficiency caused false-negative skin-test results, and topical zinc supplementation augmented antimycobacterial immune responses enough to improve diagnosis.

Tuberculosis (TB) kills >1.7 million people each year, and control is hampered by diagnostic difficulties. The TB (Mantoux) skin test measures the immune response to an intradermal injection of tuberculin (purified protein derivative [PPD]) and is important for diagnosing adult and particularly pediatric TB (*1*). However, reliability of this skin test is limited by false-negative results (*2*–*4*), especially in poorly nourished people in the resource-limited settings where most cases of TB occur (*2*). Zinc is implicated in false-negative skin tests because it modulates cutaneous reactions (*2*,*3*), because zinc deficiency is common in people with TB (*5*), and because this deficiency also suppresses antimycobacterial immunity (*4*,*5*). We therefore studied whether topical application of zinc to TB skin test sites would augment test results in Lima, Peru, a TB-endemic area in which false-negative skin-test results are frequent (*1*).

## The Study

After ethical approval and informed written consent were obtained, venous blood was collected from 50 healthy, randomly selected, adult shantytown resident volunteers. Plasma zinc concentrations were analyzed blindly by atomic absorption spectroscopy; precautions were taken against trace-metal contamination. In each study participant, the volar surface of both forearms was injected proximally with tuberculin (5 U in 0.1 mL; Aventis-Pasteur, Toronto, Canada) and distally with *Candida albicans* antigen (5 U; Hollister Stier Laboratories, Spokane, WA, USA) (*1*), totaling 4 simultaneous skin tests per person. *Candida* skin tests were conducted because, when positive, they confirm that the person can mount a cutaneous hypersensitivity reaction, which clarifies that a simultaneous negative TB skin-test result is likely to be a true negative.

In a randomized double-blind manner, skin-test sites on 1 arm were covered with 1 mL of placebo cream (Aqueous cream BP, Sandoz, Bordon, Hants, UK) and the skin-test sites on the contralateral arm were covered with zinc sulfate (*2*) dissolved in aqueous cream to a concentration of 1% elemental zinc. Each skin test site was immediately covered with an occlusive dressing (Tegaderm, 3M, London, UK). After 24, 48, and 72 h, the dressing and cream were removed, and the “ball-point-pen” test was used to measure induration. The appropriate cream was reapplied and covered after each measurement.

SPSS version 11.5 (SPSS Inc., Chicago, IL, USA) was used for statistical analysis. Except where otherwise stated, the results of skin tests are those read after 48 h. Continuous data were all normally distributed (except for food frequencies) and were therefore summarized as means (and standard errors of the mean [SEM]). Groups were compared by using *t* tests, and associations were tested by using univariate linear regression (p value and standardized coefficient shown) and in a backward multiple linear regression model with the least significant variables sequentially removed, according to the log-likelihood test.

## Conclusions

Control TB skin-test areas to which placebo cream had been applied yielded significantly smaller reactions in persons with lower concentrations of plasma zinc (p = 0.03, Table). For example, the quarter of the population with the lowest concentrations had, on average, control TB skin-test reactions that were 14 mm in diameter compared with 27 mm for the quarter of the population with the highest plasma zinc concentrations (p = 0.03). This immunologically significant zinc deficiency was frequent even though only 10% of the study population was underweight (body mass index <20 m^2^/kg).

**Table:** Population characteristics for the participants who had simultaneous skin tests with and without zinc and associations with size of tuberculosis (TB) skin-test reaction

Characteristic*	Mean (SEM)† or % (n = 50)	Association with size of control TB skin-test reaction (mm)			
		Univariate analysis		Multiple regression	
		Coefficient	p	Coefficient	p
Nutritional assessment‡§					
Body mass index (kg/m^2^)	24 (0.52) 10% underweight (<20)	0.2	0.3		
Anthropometric protein status (corrected arm muscle area; cm^2^)	36 (1.4)	0.2	0.09		
Anthropometric fat status (arm fat area; cm^2^)	14 (1.1)	0.0008	1		
Plasma zinc (mg/L; n = 49)	0.66 (0.17) 31% deficient (<0.6)	0.3	0.04	0.3	0.03
Minor incidental health symptoms	38%	0.06	0.7		
TB risk factors					
Age, y	32 (1.3)	0.2	0.2	0.3	0.02
Male	38%	0.3	0.03	0.4	0.01
Past close contact with a TB patient or past proven diagnosis of TB	42%	0.1	0.4		
Presence of Baccilus Calmette-Guérin vaccine scar(s)	86%	0.2	0.3		
Overcrowding (persons/room)	1.3 (0.08)	–0.2	0.9	0.2	0.09
Poor household ventilation (subjective assessment)	24%	–0.002	1		
Socioeconomic status					
Food spending/person/d ($US)	0.75 (0.05)	0.1	0.5		
Dirt floor throughout the home	38%	–0.06	0.7		
Home built from temporary materials	72%	0.1	0.5		
No in-house sanitation	84%	0.05	0.7		
No piped water to home	84%	–0.07	0.6		

*Variables that may influence TB skin-test reaction size are shown together with their associations with the size of the control TB skin-test reactions associated with only zinc cream application. †SEM, standard error of the mean. ‡Food frequencies: in the previous week, alcohol had been consumed a median of 1 time (median 1 U), dairy produce 3 times, fruit/vegetables 5 times, meat/fish 6 times, and rice/bread/cereals daily. §Persons with infrequent meat/fish consumption had lower protein stores on muscle anthropometry (p = 0.02), and this tended to be associated with smaller skin-test reactions, as previously reported (*1*,*2*).

**Figure 1:**
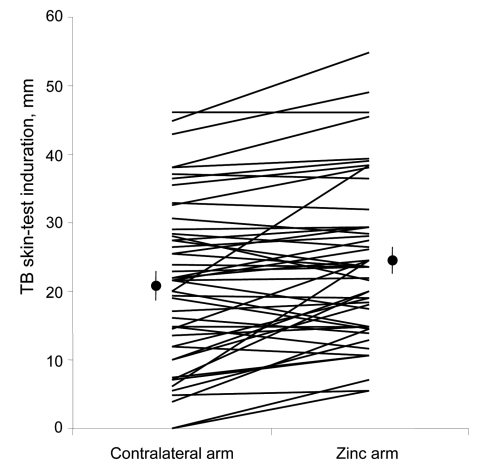
Effect of topical zinc on size of tuberculosis (TB) skin-test reaction. Each line represents the size of the 2 TB skin-test reactions for 1 person with placebo cream (on the left of the graph) and topical zinc cream (on the right). Circles show the mean TB skin-test reaction size without and with topical zinc, with standard error bars.

**Figure 2:**
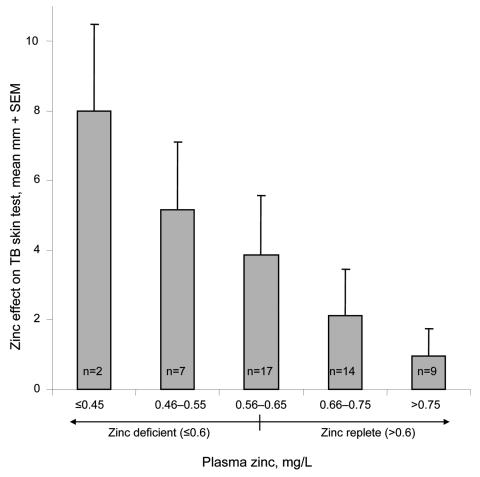
Association between plasma zinc concentration and response to topical zinc. The association is shown between plasma zinc concentration and the magnitude of augmentation of the purified protein derivative skin test with topical zinc. The normal range of plasma zinc (>0.6 mg/L) is also indicated.

The TB skin-test results were read in a blinded manner, and reactions to tests with zinc cream applications were an average of 32% larger than the contralateral control skin test reactions with placebo cream applied (Figure 1; p<0.001). TB skin-test results were considered positive if the average diameter of induration was >10 mm 48 h after injection, according to national policy. Skin tests with zinc applications were significantly more likely to have positive results than the simultaneous contralateral control skin tests with placebo cream applications (94% zinc vs. 76% placebo, p = 0.01). Topical zinc caused greater TB skin-test augmentation in persons with lower plasma zinc concentrations (correlation coefficient [*R*] = 0.3, p = 0.05), and persons with absolute plasma zinc deficiency (<0.6 mg/L) had significantly greater zinc-mediated augmentation (Figure 2, p = 0.02). Zinc cream had no effect in persons with normal plasma zinc concentrations. Thus, zinc cream significantly augmented TB skin-test reactions only in persons deficient in zinc (Figure 2).

TB skin tests are usually read 48 h after PPD injection; however, in this study, additional readings were done at 24 and 72 h, at which time results, similar to those at 48 h, demonstrated that zinc cream caused larger TB skin-test reactions (both p<0.02) and more positive test results (both p<0.006). Furthermore, at 72 and 48 h, this zinc-mediated augmentation was greater in zinc-deficient persons (p = 0.006), although the similar trend at 24 h was not statistically significant. The magnitude of skin-test augmentation with zinc cream, the plasma zinc concentration, and the anthropometric measures had no significant associations with other studied factors except as noted in the Table.

The effect of zinc on the *Candida* skin-test results was similar to the effect on the TB skin-test results. Specifically, the *Candida* skin-test reactions with zinc cream were an average of 23% larger than the contralateral skin-test reactions associated with placebo cream applied in a double-blind manner (p<0.001). *Candida* skin-test results were considered positive if the average diameter of induration was >5 mm (*1*), and 78% of *Candida* skin-test results associated with zinc application were positive, compared with 64% with placebo cream (p = 0.01). Zinc-mediated TB and *Candida* skin-test augmentation were associated (*R* = 0.3, p = 0.05), but zinc-mediated *Candida* skin-test augmentation was not significantly associated with any of the variables in the Table.

The control skin tests with placebo cream yielded larger reactions (p = 0.005) and were more likely to be positive (p = 0.005) than concurrent skin tests on 97 other randomly selected healthy volunteers in the same community (data not shown). These 97 skin tests were each performed on 1 arm only, and no creams were applied; the mean diameter of induration was 16.4 mm (SEM 1.9 mm), and 52% were TB skin-test positive (>10 mm). Although the design of this part of the study prevented blinded assessment, this comparison implies that in the 50 persons with simultaneous bilateral skin tests, zinc absorption through the skin may have partially augmented the contralateral control skin-test reactions to which placebo cream was applied. Although the local direct effect at the site of zinc cream application was significantly greater, systemic zinc absorption may have caused the effect of zinc cream to be underestimated; this hypothesis is being further investigated.

Zinc status is difficult to reliably assess (*2*–*4*). However, low plasma zinc significantly predicted small skin-test reactions and the magnitude of the immunologic response to topical supplementation. Thus, zinc cream had a specific effect, namely, reversing the skin-test suppression of zinc deficiency. Topical zinc supplementation applied to the arms of persons who received TB skin tests therefore identified persons in whom zinc deficiency was sufficient to suppress antimycobacterial immune responses and quantified the immune-potentiating effect of supplementation. This approach may similarly facilitate the therapeutic evaluation of other micronutrients and immunomodulatory compounds.

Zinc cream had no effect in persons with adequate concentrations of zinc; however, persons who were deficient in zinc but otherwise apparently healthy had suppressed skin-test reactions, despite the absence of frank malnutrition; this suppression was reversed by the application of zinc cream. Thus, zinc cream application corrected false-negative TB skin-test reactions caused by zinc deficiency, allowed more sensitive diagnosis, and hence facilitated appropriate treatment of latent TB infection. Topical zinc is a simple and relatively inexpensive method of enhancing reliability of the established TB skin test for diagnosing this neglected disease. This has public health implications because the TB skin test is central to the problematic area of diagnosing TB infection and disease, especially in children who are prone to both zinc deficiency (*2*,*4*) and false-negative skin-test results (*1*). Therefore, the zinc-mediated augmentation of TB skin testing that we have demonstrated may facilitate the diagnosis of adult and pediatric cases of TB in regions where micronutrient deficiency is prevalent.

In conclusion, this study demonstrated that physiologically significant subclinical zinc deficiency was common in this population, that low plasma zinc predicted negative TB skin-test results, and that topical zinc supplementation augmented local antimycobacterial immune responses sufficiently to reverse this anergy of micronutrient deficiency.
